# COVID-19 Induced Coagulopathy (CIC): Thrombotic Manifestations of Viral Infection

**DOI:** 10.1055/s-0042-1744185

**Published:** 2022-03-10

**Authors:** Swati Sharma, Aastha Mishra, Zahid Ashraf

**Affiliations:** 1 Deptartment of Biotechnology, Jamia Millia Islamia, New Delhi, India; 2 CSIR-Institute of Genomics and Integrative Biology, Delhi, India

**Keywords:** covid-19, coagulopathy, thrombosis, inflammation

## Abstract

Coronavirus disease 2019 (COVID-19) is caused by the severe acute respiratory syndrome coronavirus 2 (SARS-CoV-2) and may result in an overactive coagulative system, thereby resulting in serious cardiovascular consequences in critically affected patients. The respiratory tract is a primary target for COVID-19 infection, which is manifested as acute lung injury in the most severe form of the viral infection, leading to respiratory failure. A proportion of infected patients may progress to serious systemic disease including dysfunction of multiple organs, acute respiratory distress syndrome (ARDS), and coagulation abnormalities, all of which are associated with increased mortality, additionally depending on age and compromised immunity. Coagulation abnormalities associated with COVID-19 mimic other systemic coagulopathies otherwise involved in other severe infections, such as disseminated intravascular coagulation (DIC) and may be termed COVID-19 induced coagulopathy (CIC). There is substantial evidence that patients with severe COVID-19 exhibiting CIC can develop venous and arterial thromboembolic complications. In the initial stages of CIC, significant elevation of D-dimer and fibrin/fibrinogen degradation products is observed. Alteration in prothrombin time, activated partial thromboplastin time, and platelet counts are less common in the early phase of the disease. In patients admitted to intensive care units (ICUs), coagulation test screening involving the measurement of D-dimer and fibrinogen levels, has been recommended. Prior established protocols for thromboembolic prophylaxis are also followed for CIC, including the use of heparin and other standard supportive care measures. In the present review, we summarize the characteristics of CIC and its implications for thrombosis, clinical findings of coagulation parameters in SARS-CoV-2 infected patients with incidences of thromboembolic events and plausible therapeutic measures.

## Introduction

Coronavirus disease of 2019 (COVID-19) is a viral infection caused by the severe acute respiratory syndrome coronavirus 2 (SARS-CoV-2). 1 2 3 4 Because of its highly contagious nature and global spread, it was declared a pandemic by WHO since early March 2020. 5 SARS-CoV-2 comprises of positive-sense single-stranded RNA genome harboring a surface glycoprotein known as spike protein, or S proteins. These proteins are believed to be responsible for the tropism toward the specific receptors present on the cell surface of the target organism. The virus specifically targets respiratory epithelium via angiotensin converting enzyme 2 (ACE2) receptor, thereby making its entry into the host cells. 6 ACE2 receptors are highly expressed in many different cell types, including lung alveolar cells, cardiac myocytes, and vascular endothelium. 7 Primarily, SARS-CoV-2 is transmitted by inhaling viral particles, facilitating their entry into the respiratory tract. 1 Additionally, the virus disseminates through fomite transmission, depending on the different surfaces and varied time intervals in which the virus can persist. 8 The initial presentation of COVID-19 overlaps with that of other viral syndromes and includes fever, cough, fatigue, shortness of breath, headache, diarrhea and myalgias. 9 10 11 However, the respiratory distress syndrome accompanying COVID-19 may differ from traditional acute respiratory distress syndrome caused by other viruses of the same family. Despite the extremity of hypoxemia in this infection, there is ongoing lung damage, marked by increased respiratory compliance and shunt fraction along with heightened recognition of systemic features of a hypercoagulable state. The alteration in coagulation parameters caused by SARS-CoV-2 infection can be termed ‘COVID-19 induced coagulopathy’ (CIC), which may cause various adverse cardiovascular complications, ultimately leading to death in some patients. 12 13 CIC is different from the classic DIC observed in the case of sepsis. Coagulation changes in the COVID-19 patients though mimic that of DIC but are not identical. In the patients with COVID-19 infection, a strong local pulmonary thrombotic microangiopathy along with the direct endothelial cell infection by the viral particles is induced that inflict the coagulopathic response. The elevated plasma D-dimer level is the most remarkable abnormal coagulation feature in the case of severe COVID-19 patients. Whereas, in sepsis-associated DIC, a more profound thrombocytopenia is reported. In addition, DIC patients exhibit much lower levels of clotting factors and significant decrease in plasma concentrations of coagulation inhibitors like antithrombin and protein C. These features are not observed in CIC. 14

Furthermore, patients who have certain co-morbidities, in particular as associated with cardiovascular disease, are speculated to have the worst prognosis among COVID-19 patients. Amidst the cardiovascular comorbid conditions, patients with diabetes are more likely to have severe infection. Other comorbidities, such as chronic obstructive pulmonary disease, liver and renal diseases, have also been associated with poor disease progression. Several studies, as well as case reports, suggest patients with severe infection present with coagulation dysfunction, as evident from a rise in blood D-dimer, lactate dehydrogenase and total bilirubin levels. Standard coagulation parameters, like partial thromboplastin time (PTT) or activated partial thromboplastin time (aPTT) and platelet count show slight or no change in the initial presentation of the disease. Only 5% of the reported cases exhibit prolonged PT and 6% prolonged aPTT as an initial manifestation of the disease. 15 However, with progression of disease and severity there is progressive prolongation of PT, whereas aPTT appears to remain largely unchanged in non-fatal COVID-19 cases vs fatal cases, with no significant correlation with disease severity or mortality. 16 Initial clinical studies showed patients with elevated level of troponin indicative of cardiac injury, but due to lack of imaging data such as cardiac magnetic resonance imaging (MRI) or echocardiography, the mechanism remained unclear. Significantly higher deaths are reported in patients with cardiac injury (51.2%) as compared with those without any cardiovascular complication (4.5%). 17 Also, there is speculation that the virus may act through direct cell damage upon ACE2 receptor binding, causing a systemic inflammatory response with an arising cytokine storm, destabilizing coronary plaques, and aggravating hypoxia, which represent risk factors for thrombotic events. 16

**Fig. 1:**
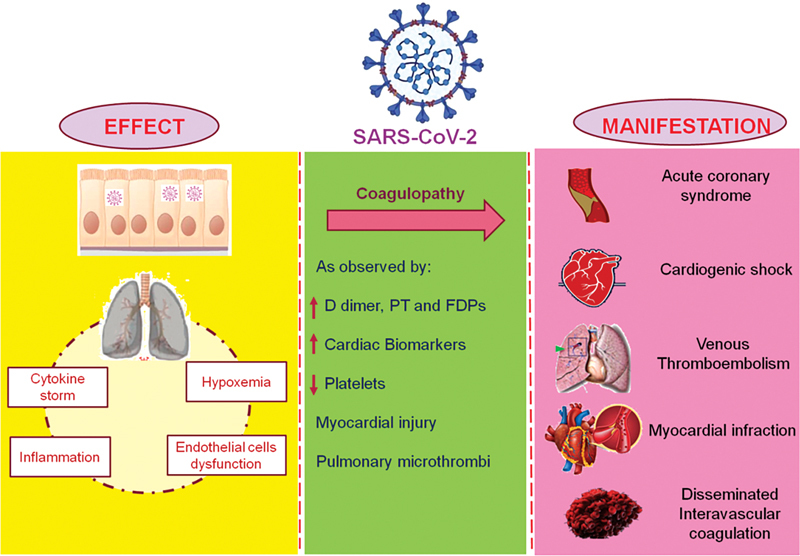
Depiction of effect and manifestation of COVID-19 and associated cardiovascular implications.

SARS-CoV-2 may have a direct effect on hemostasis or it may act indirectly by creating pathological effects such as hypoxia and inflammation that may predispose COVID-19 patients to thromboembolic events. Preliminary data are suggestive that hemostatic abnormalities such as CIC, occur in COVID-19 patients. 16 18 Also, the heightened inflammatory response causing cytokine storm, severe illness, and underlying traditional risk factors may all predispose an infected patient to thrombotic events or other cardiovascular manifestations ( Fig. 1 ). 19 Therefore, an understanding of CIC mediated thrombotic complications in COVID-19 is of utmost importance for better management of the severe cases of the infection. In the present review we summarize all the interpretations of the characteristics of CIC till date with its implications for thrombosis and its management. We also present clinical findings related to coagulation parameters in infected patients with incidences of thromboembolic events and probable therapeutic measures for the effective management of the disease.

## Thrombotic Incidences in COVID-19 Patients Exhibiting CIC

**Fig. 2:**
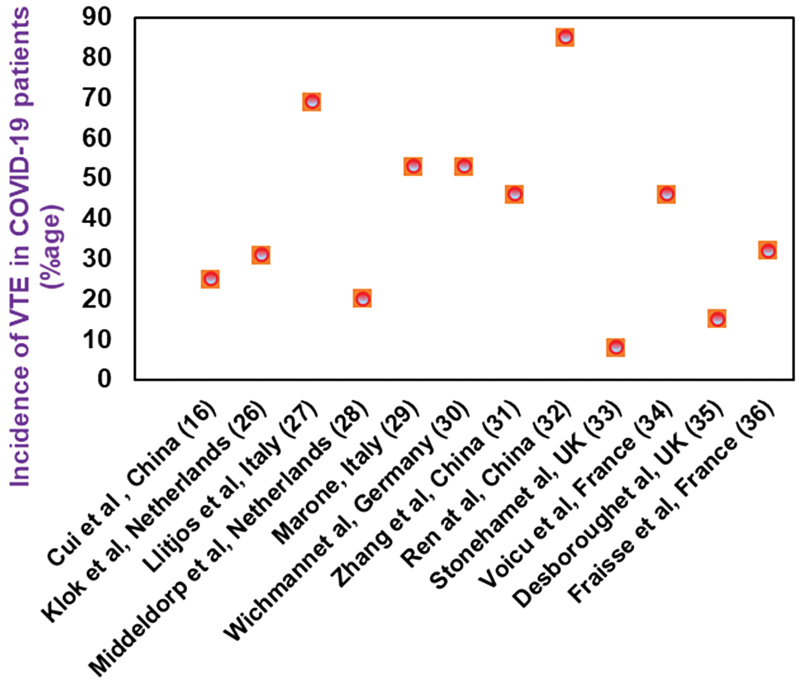
Thromboembolic complications in COVID-19 patients. 16 26 27 28 29 30 31 32 33 34 35 36

Up to November 2021, more than 253.1 million cases of COVID-19 have been reported globally accounting for ∼5.1 million deaths. 20 There are also reports of asymptomatic carriers 15 16 21 22 ). The association of abnormal coagulopathy with infection was first observed in the initial reports from Wuhan, China showing 6% elevation in aPTT, 5% in PT and 36% in D-dimer in the first 99 patients admitted to a hospital. 23 These patients showed increased inflammatory markers, including interleukin-6 (Il-6), erythrocyte sedimentation rate (ESR), and C-reactive protein (CRP). 15 The initial reports were suggestive of high risk of thromboembolic complications in these patients. With the progression of the pandemic, numerous other reports have revealed prolonged PT, high D-dimers, along with high levels of fibrinogen and high CRP, lymphocytopenia and mild thrombocytopenia. 8 18 A systemic review consisting a total of 36 studies including the patients requiring ICU placement, presented the pooled incidence rate of 28% (95% CI, 22–34%) for venous thromboembolism (VTE). 24 Micro-thrombosis formation was noted in 80% of lung autopsies from fatal COVID-19 cases. 25 High incidence of VTE (31%) resulting in complications such as pulmonary embolism (PE) (80%), as well as arterial thrombosis (3.7%), has been reported in COVID-19 patients by Klok et al. 26 In Fig. 2 , 16 26 27 28 29 30 31 32 33 34 35 36 we have summarized the incidences of venous thromboembolism (VTE) reported in COVID-19 patients, according to different studies.

## Molecular Mechanism Resulting in CIC Mediated Thrombosis

### Endotheliitis and Hyperinflammation

**Fig. 3:**
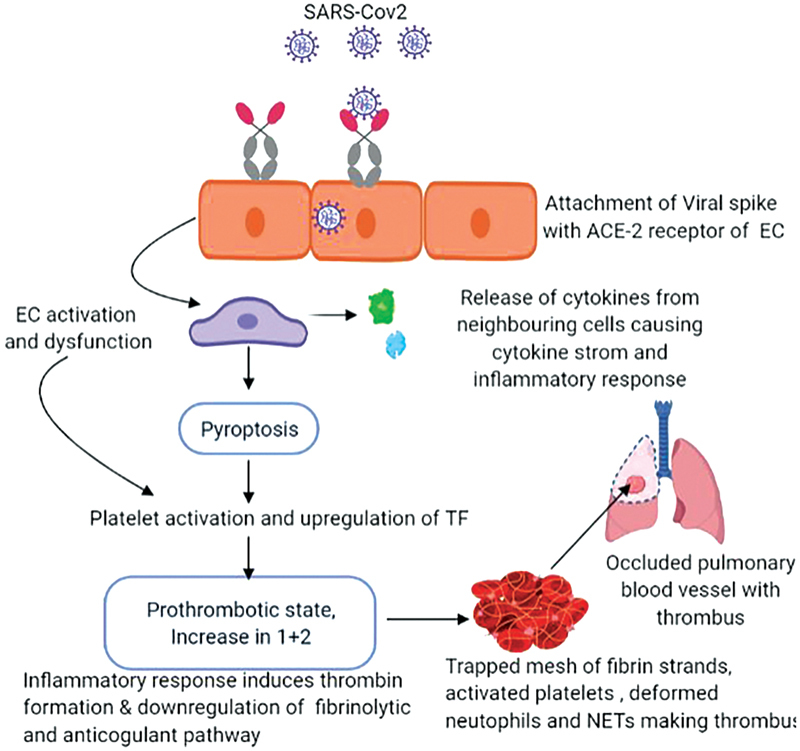
**Mechanism of CIC induced Thrombosis:** The viral particles enter the host through ACE-2 receptors found on endothelial cells (EC) of respiratory pathway resulting in endothelial activation and dysfunction that leads to pyroptosis and cytokine storm. This in turn causes platelet activation and upregulation of tissue factor (TF) and fragment (1 + 2). The spiked inflammatory response along with downregulation of anticoagulant and fibrinolytic pathway leads to formation of thrombus.

Endotheliitis and hyperinflammation initiates CIC. The main site of viral infection in humans is lung alveolar type II epithelium, where the spike protein S1 attaches itself to the ACE-2 receptor. 6 9 The virus replicates in the infected cells causing inflammatory response resulting in pyroptosis. The receptors of innate immunity like TLR (Toll-like receptors) detect danger-associated molecular patterns (DAMPs) such as ATP and DNA released after pyroptosis and evoke intense inflammatory responses along with release of proinflammatory chemokines and cytokines from neighboring cells 37 ( Fig. 3 ). Also, TLR-7 and TLR-8 recognizes the multiple single-stranded RNA fragments of SARS-CoV-2, thus contributing to innate immune hyper activation and causing a cytokine storm. 38 Thus, upon infection, the vascular endothelium, which is a guardian of vascular integrity, gets hijacked by the virus and then exposed to the milieu of proinflammatory cytokines elicited by the innate immunity. 39 All the studies conducted have hypothesized that the entry of the virus via endothelial-expressed ACE-2 paves the way by which the virus can enter and infect other tissues.

ACE-2 is also highly expressed in various extrapulmonary sites, including blood vessels, heart, kidney, and intestine. 40 41 Clinical data from infected patients have revealed increased levels of IL-6, IL-1β, MCP-1 (monocyte chemo-attractant protein 1), MIP (macrophage inflammatory protein), IFN (interferon)-γ, and IP10 (CXCL10). 1 Disruption of endothelial function and integrity by these proinflammatory cytokines leads to release of von Willebrand factor (VWF), upregulation of adhesion molecules such as ICAM (intercellular adhesion molecule)-1, integrin αvβ3, P- selectin, E-selectin and endothelial tissue factor (TF, CD142). 42 43 The sudden activation of endothelial cells causes elevated VWF antigen (VWF:Ag) levels. The function of VWF is mediated through proteolysis guided by von Willebrand factor-cleaving protease, which is also known as a disintegrin and metalloprotease with thrombospondin type 1 motif, member 13 (ADAMTS-13). It is hypothesized that in the case of COVID-19 infection, ADAMTS-13 regulation of VWF multimer distribution is impaired resulting in procoagulant state. 44 Ilaria et al reported a significant change in the VWF-ADAMTS13 axis in infected patients, with an increase in VWF:Ag to ADAMTS13 activity ratio, which is strongly associated with disease severity. This imbalance in the ratio of VWF level to ADAMTS13 increases the hypercoagulable state of COVID-19 patients and thereby risk of micro-thrombosis. 45 Thus, this cascade of events cause endothelium to adopt a procoagulant phenotype, which is then supportive of platelet and leukocyte recruitment and marks the initiation of CIC. 46 47

IL-6, a crucial cytokine that is markedly increased in severe COVID-19, is an activator of coagulation that induces TF expression along with increased production of fibrinogen and platelets. 48 49 50 Increased Angiotensin II (ATII) expression, along with proinflammatory cytokines and antiphospholipid antibodies are responsible for TF activation and overexpression in the infected patients. Thus, it is likely that the overexpression of TF in COVID-19 is related to the pathogenesis of the disease. 51 The researchers have looked for correlation between TF expression and the severity of COVID-19 and found that TF expression both in monocytes and extracellular vesicles is associated with severity of disease. 52 53 This is because TF can increase its expression during localized hypoxia of lungs in the case of COVID-19 infection and generate a thrombotic phenotype, which is observed to be regulated by extracellular RNA activated Toll-like receptor 3-activated protein 1 signaling. 54 This in turn initiates extrinsic cascade of coagulation and spiked inflammatory response. This inflammation further causes endothelitis in CIC.

These observations are in alignment with clinical autopsy of COVID-19 patients, as viral inclusion within the endothelial cells is seen in sections of lungs and kidney, thus highlighting the ability of virus to cause endothelial injury after infection. These findings are augmented with histological evidence of endothelial inflammation and cell death by viral load. 39 Together, these findings suggest a direct role of SARS-CoV-2 in promoting systemic microvascular dysfunction causing a prothrombotic milieu. 48

### Role of Platelets CIC Mediated Thrombosis

In severe cases of COVID-19, one of the common findings is thrombocytopenia. A recent meta-analysis reveals association of thrombocytopenia with 5-fold increase in risk of severe infection. 49 The recent studies demonstrated that severe COVID-19 cases often have cardiovascular comorbidities suggestive of thrombocytopenia or platelet apoptosis as a major contributor of virus pathogenesis. 50 55 Other molecular events involved in SARS-CoV-2 pathogenesis, like inflammation, hypoxia, immune system activation, endothelial activation and dysfunction can lead to platelet apoptosis as well as activation, resulting in increased thrombotic events. 55 56 57 58 Patient autopsies from those with severe SARS-CoV-2 infection show platelet-rich thrombotic microangiopathy. 59 60 61 The presence of arterial and venous thrombus through-out the lungs is suggestive of CIC influenced platelet hyperactivation that in turn increases the severity of the disease by exacerbating inflammation. Platelets express a wide repertoire of adhesion as well as immune receptors, like FcγRIIa, CXC/CCL receptors and TLRs along with an ability to engulf virions. SARS-CoV-2 has been demonstrated to activate platelets via TLR-7 and the FcγRIIa receptors, which evokes an array of functional responses that support thrombus formation, including formation of platelet leuckocyte aggregates, platelet degranulation, and so on. 62 63 64 65 Activated platelets expressing CD40L and P-selectin interact with neutrophils and release α-granules along with complement C3 and different cytokines like CC-chemokine ligand 2 (CCL2), CCL3, CCL7, IL-7, IL-8, IL-1β. 66 67 These findings correlate with the clinical data of COVID-19 patients, as the level of these cytokines have been reported to be significantly elevated in these patients compared with healthy controls. 68 Previous studies with dengue virus show that platelets release IL-1β that results in increased permeability. 69 The release of IL-1β and other cytokines by platelets in response to SARS-CoV-2 possibly causes COVID-19 associated ARDS. As seen in COVID-19 pathology, parallel to cytokine release, another important event in ARDS, is recruitment of neutrophils to the pulmonary vasculature. 70 The molecular mechanism by which activated platelets binds to neutrophils and lead to rolling of platelet-bound neutrophils on the endothelium, is termed as ‘secondary capture’. This plays a pivotal role in the initiation of immune-thrombosis. 71 The transmigration of the platelets to the alveolar lumen is mediated by binding of activated platelets to neutrophils and results in the formation of edematous lungs, which leads to further platelet activation. 70 This series of events leads to formation of neutrophil extracellular traps (NETs). Recent reports suggest that NETs trigger thrombo-inflammation in COVID-19 patients, 70 causing vascular thrombosis and high risk of mortality. 72 Taken together, platelet activation along with apoptosis are contributors to the severity of pathology of COVID-19, which includes thrombosis and the cytokine storm.

Along with mediating T cell function and leukocyte aggregation, platelets also increase B cell immunoglobulin (Ig) G1, IgG2, and IgG3 production. 73 This finding is significant in the light of COVID-19 as formation of antiphospholipid antibodies, like anti-β2 glycoprotein IgG antibodies, are associated with thrombosis in patients with COVID-19. 74 A cohort studied from France has demonstrated that a large percent of severe COVID-19 patients exhibits a detectable lupus anticoagulant. 75 However, it still remains to be established whether the presence of antiphospholipid antibodies in SARS- CoV-2 patients plays a role in infection-associated thrombosis or represents merely an association. Nevertheless, deciphering the ways platelets modulate the adaptive immune response to COVID-19 and the role played by adaptive immunity mediating thrombosis will be crucial in uncovering novel therapeutic strategies to combat the disease.

## Prediction of CIC Severity and Occurrence of Thrombotic Events

### Analysis of Coagulation and Inflammatory Parameters

There are 6 baseline coagulation tests that should be performed to measure the extent of CIC in infected patients. These are PT, aPTT, D-dimer, fibrinogen, fibrin degradation products (FDP) and platelet count. These tests represent traditional tests used to help assess bleeding or thrombotic risk. Additional laboratory measures to evaluate CIC associated inflammation are C-reactive protein (CRP), erythrocyte sedimentation rate (ESR), ferritin, procalcitonin, and high-sensitivity cardiac troponin. Inflammatory markers such as CRP, ESR are elevated in COVID-19 patients. These markers are also associated with the occurrence of thrombosis. We discuss below the potential utility of the 6 baseline coagulation tests that can be used to help access the severity of disease and predict the future course of medical intervention.

- PT: In the initial stage of the disease, PT is normal or near-normal in most COVID-19 patients. Only 5% cases showed prolonged PT during the initial manifestation of the disease. 16 However, there is significant change in the PT profile in critically ill COVID-19 patients having CIC. 70 71 Critically ill patients show an average increase 1.9s in PT as compared with non-fatal or critical cases. Also, as the severity of the disease progress, ∼48% of critically ill patient exhibit marked and progressive increase in PTs. 19 Thus, an upward trending PT can add clinical evaluation in monitoring progression of the disease, in particular fatal or critical cases. That is, progressive increase in PTs can be considered a warning sign and a predictor of increased risk of mortality.
- aPTT: the aPTT profile is often unaltered in COVID-19 patients, and only a fraction (6%) of patients shows prolonged aPTT. 16 There is no significant difference in the average aPTT between fatal and non-fatal cases of infection. Also, no significant correlation was observed between aPTT and disease severity or mortality. 19 Therefore, the aPTT cannot be considered as reliable indicator of CIC and disease progression in COVID-19. Also, the aPTT cannot be associated with prevalence of VTE in COVID-19 cases.
- D-dimer: quantitative D-dimer is used routinely as a biomarker for exclusion/diagnosis and prediction of recurrence of VTE. 76 77 36% of reported cases have showed elevated levels of D-dimer (approximately, 0.9 mg/L). 12 16 An elevated D-dimer level is observed in fatal cases as compared with non-fatal cases (mean level of 2.4 vs 0.5 mg/L) and is inversely correlated with the survival of patient. 12 78 Additionally, non-survivors have shown steady and progressive elevation in D-dimer level, whereas in COVID-19 survivors the levels remain constant or improves. 79 Endothelial dysfunction caused by viral entry results in elevated levels of D-dimer along with thrombin and FDPs, inflammation and hypoxia resulting in CIC which can lead to pulmonary congestion mediated by thrombosis. 79 These findings suggest D-dimer to be highly prognostic in COVID-19. Evaluation of D-dimer level may also aid in identifying patients that could benefit from anticoagulant therapy.
- Fibrinogen: Most COVID-19 patients show elevated (4.55 g/L) fibrinogen in the initial stage of the disease. The degree of elevation correlates with increased IL-6 levels but not with mortality. 17 79 Il-6 is an important mediator of inflammation and in COVID-19, inflammation precedes coagulation. As the disease progress, there may be a progressive decrease in the fibrinogen level, and at a late course of the disease is strongly associated with mortality. Approximately 29% of critically ill patients had fibrinogen <1 g/L, but this happens at the last stage of the disease. 17 Thus, fibrinogen levels cannot be used for prognosis of the initial phase of COVID-19, but progressive deterioration may reflect a poor prognosis.
- FDPs: FDP levels are relatively constant in both mild cases and initial presentation of the disease. However, critically ill patients admitted to ICU showed significantly higher levels of FDP. The approximate levels of FDP in survivors are 4 μg/mL vs 7.6 μg/mL in the non-survivors. The elevation of D-dimer is followed by elevation of FSP. Though D-dimer is more sensitive marker for coagulopathy during the initial phase of the disease, FSP can also be considered prognostic for the COVID-19 infection as progressive elevation of FSP level correlates with mortality 17
- Platelet count: Platelet count is mostly unaltered or slightly reduced in most cases of COVID-19 and thrombocytopenia is reported in only 12–36% of total cases. 12 16 19 Platelets play a crucial role in progression of coagulopathy and in the development of thrombosis (explained in the earlier section of the review). Severe thrombocytopenia correlates with progression of disease as more than 55% of critically ill COVID-19 patients have a platelet count of <100 × 10 ^9^ /L.

## Therapeutic Perspectives

**Fig. 4:**
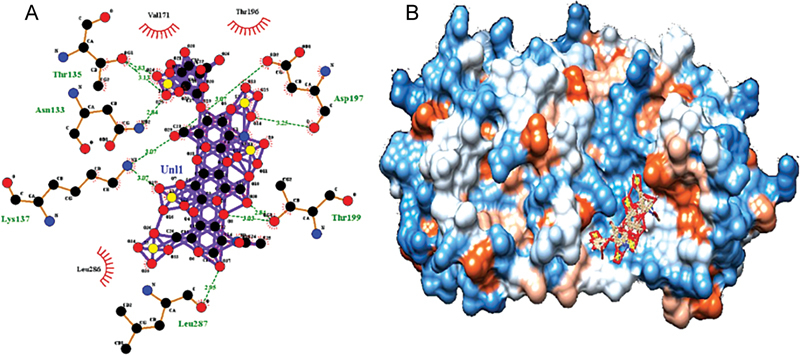
**Binding of heparin with COVID-19 main protease** : ( **A** ) **.** Ligplot view of blind docking of main protease of COVID-19, (PDB: 6y2e) with heparin (Pubchem: 772). For a given protein-ligand docked PDB file, LigPlot automatically generates schematic diagrams as represented by hydrogen bonds and by hydrophobic contacts. Green dashed lines between the atoms involved represent the hydrogen bonds and an arc with orange spikes pointing toward the ligand atom they contact with represents the hydrophobic contacts. ( **B** ). Hydrophobicity surface view of protease bound to heparin: image as created by chimera represents “hydrophobicity surface” that is dodger blue for the most hydrophilic, to white, to orange red for the most hydrophobic.

The lack of specific therapy against SARS-CoV2 and significant association of thrombotic complications with severe COVID-19 has led to exploration of anti-thrombotics as a choice of drug for infected patients. Antithrombotic drugs having anti-inflammatory and/or antiviral properties are also under clinical trial. Anticoagulants are first choice of antithrombotic therapy. VTE prophylaxis with either low molecular weight heparin or unfractionated heparin is commonly applied to COVID-19 patients. 79 80 Initial clinical reports suggested that administration of low molecular weight heparin reduces the death rate in COVID-19 patients with an elevated sepsis-induced coagulopathy score or an elevated D-dimer level. 81 82 Heparins, apart from being effective anticoagulants seem to have pleiotropic effects by way of their ability to bind to DAMPs, such as HMGB-1, and proinflammatory cytokines that may be additionally therapeutic in the context of viral infection, including anti-inflammatory effects. 82 83 Recent findings are suggestive of other links between heparin and SARS-CoV-2, for example that the antiviral properties of heparin can be attributed to its ability to bind with the spike protein of the SARS-CoV-2 virus directly. 84 Similar results have been shown after docking of a major protease (PDB id 6y2e) of COVID-19 with heparin. It showed binding with affinity -11.8 Kcal/mol ( Fig. 4 ). But the use of heparins in COVID-19 cases is limited to individual case reports because of the potential for heparin therapeutics to cause heparin-induced thrombocytopenia. 85

**Fig. 5:**
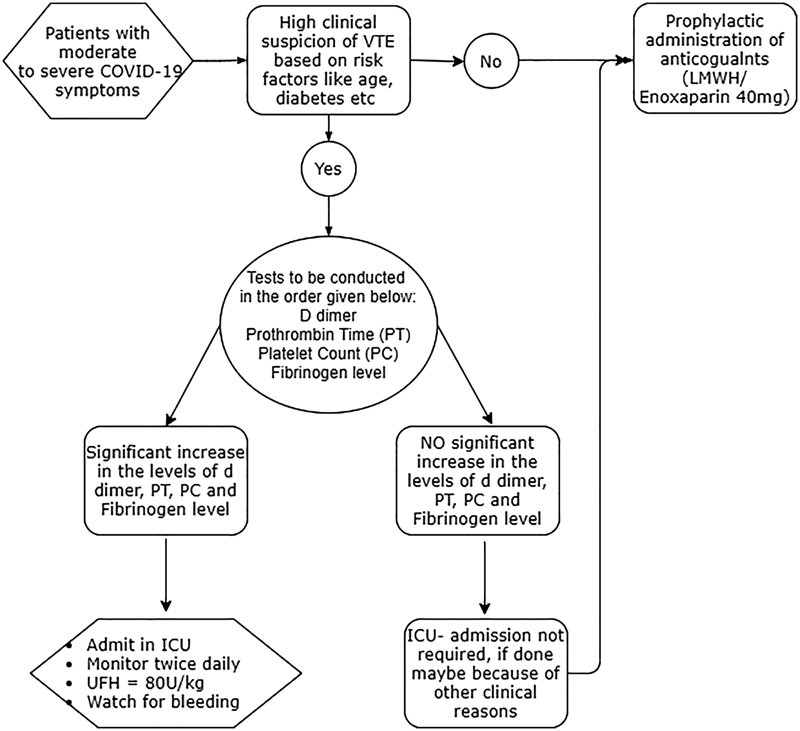
**Flow chart for management of COVID-19 associated VTE** . Abbreviations: Prothrombin time (PT), Platelet count (PC), Intensive care unit (ICU), Low molecular weight heparin (LMWH), Un-fractioned heparin (UFH).

Another therapeutic approach is the use of inhibitors of the contact factor activation pathway. Inhibition of thrombo-inflammatory response by inhibition of FXII also appears to be an attractive therapeutic target. Activation of FXII by negatively charged molecules like NETs and platelet derived PolyP initiates activation of contact pathway. The level of NETs and PolyP is reported to be upregulated in severe COVID-19 patients. 86 Activation of contact pathway causes thrombin generation and bradykinin activation. This in turn causes downstream complement activation and production of inflammatory cytokines. 87 It has been demonstrated in animal models of thrombosis that inhibition of FXIIa gives protection from occlusive thrombus formation without impeding hemostasis. 88 The potential safety of FXII inhibitors is attributed to the fact that administered individuals display no bleeding disorder, and there is no reported effect on immune function. 89 Therefore, a FXII inhibitory antibody can be a promising novel combination of antithrombotic and anti-inflammatory drugs that can be used in COVID-19. Another therapeutic approach that may help in COVID-19 includes fibrinolytic agents such as tPA (tissue-type plasminogen activator). This interest in tPA has emerged from the concept that ARDS is marked by significant local inflammatory reaction along with a hypofibrinolytic state. As described earlier, severe COVID-19 may cause ARDS that can result in microvascular thrombosis, extensive thrombin generation and fibrin formation, because of mass upregulation of inflammatory cytokines and leukocytes. 90 In reports of its use in severe infection case, an initial improvement was observed after tPA infusion. 91 92 93 Antiplatelet therapies are also potentially promising in managing COVID-19 associated thrombosis. The majority of clinical findings associating antiplatelet therapy with improved outcomes relates to aspirin. 94 Recent experimental data has shown that aspirin prevents neutrophil mediated microvascular thrombosis and intravascular coagulation by targeting platelets in a mouse model of bacterial sepsis. 95 Still, the effect of aspirin in assisting in COVID-19 symptoms remains to be established. Various other antiplatelet therapeutics, including dipyridamole and nafamostat, are currently being accessed for their potential to reduce the severity of COVID-19. Recently it has been reported that dipyridamole suppresses SARS-CoV-2 replication in vitro. The earlier studies with same drug suggest that use of adjunct dipyridamole may improve the clinical course in severe cases of COVID-19. 96 Nafamostat, a serine protease inhibitor that is marketed in Asia for the treatment of DIC and pancreatitis is being evaluated for its role in helping with COVID-19 associated coagulopathy because of its antiplatelet effect. 97 Fig. 5 depicts a flow chart for management of COVID-19 associated VTE. With all the available evidence-based guidelines for the treatment of COVID-19, it can be said that the current therapies are inadequate as they do not substantially reduce morbidity and mortality. However, with the new molecular mechanistic insights emerging from the concerted efforts of biomedical research, more opportunities will arise to identify potentially safe and effective therapeutic strategies for COVID-19.

## Conclusion

This pandemic has jeopardized the security and stability of the entire human race for an unknown future duration, plunging it into uncharted territory, leaving all of us feeling powerless in the face of an infectious and invisible threat. The emergence of the virus has presented unprecedented healthcare and economic challenges globally. The decoding of pathological manifestations of this virus becomes paramount in the management of this disease. A crucial clinical feature of severe COVID-19 infection is presence of prothrombotic milieu, which is associated with increased rates of arterial, venous and microvascular thrombosis, along with adverse clinical outcomes. Emerging evidence from the experimental findings reveal that SARS-CoV-2 can infect endothelial cells and invoke immune response. This results in activation of inflammatory pathways causing endothelium dysregulation, leukocyte activation, NET generation, complement deposition, and thrombocytopenia. All these events together conspire to unleash a prothrombotic state (immune-thrombosis) that leads to significant thrombotic complications. Nevertheless, uncovering the precise mechanisms of SARS-CoV-2 infection and associated thrombotic complications is required to infer novel therapeutic approaches. Thus, in the context of this pandemic, the intersection of adaptive and innate immunity with inflammation and thrombosis has been thrust into the global spot-light. With the growing experimental evidences and understanding of thrombotic complications in SARS-CoV-2 infection, more rigorous diagnostic approaches resulting in early detection of thrombotic events can be developed. Also, the use of antithrombotic therapy for the prevention and treatment of COVID-19-associated thrombosis will help in improving disease outcomes in COVID-19 patients.
